# Special Issue “Physiology and Pathophysiology of Placenta 2.0”

**DOI:** 10.3390/ijms25094586

**Published:** 2024-04-23

**Authors:** Giovanni Tossetta

**Affiliations:** Department of Experimental and Clinical Medicine, Università Politecnica delle Marche, 60126 Ancona, Italy; g.tossetta@univpm.it

We are pleased to present this Special Issue of the *International Journal of Molecular Sciences*, entitled “Physiology and Pathophysiology of Placenta 2.0”.

The development of a normal and functional placenta is essential for correct fetal development. Thus, placentation is a key process that is tightly regulated. The placenta is a temporary but essential organ with different and fundamental functions during pregnancy [1,2,3]. Placental development is regulated by several factors such as growth factors, hormones, and many other types of molecules that control placental cell proliferation, differentiation, migration, and invasion [4,5].

This multitude of factors involved in placental development influence (either activating or inhibiting) several signaling pathways involved in the expression of specific genes necessary for a successful pregnancy. The importance of normal placental development becomes evident when this process is impaired, leading to severe pregnancy complications [6] such as preeclampsia (PE), fetal growth restriction (FGR) [7], gestational trophoblastic diseases (GTD) [8], preterm delivery [9], and gestational diabetes mellitus (GDM) [10]. Pregnancy can also be altered by exogenous agents such as bacteria, viruses, chemicals, and natural compounds that can impair the normal placental functions, thus altering pregnancy outcome. Many of the pregnancy complications previously mentioned are associated with an increase in maternal and fetal mortality and morbidity, and can cause life-long health complications for both mother and child.

Important signaling pathways such as TGFβ/SMAD, PI3K/AKT/mTOR, and JAK/STAT have been reported to be impaired in a number of pregnancy complications such as PE, GDM, and FGR [11,12,13]. Furthermore, normal placental development can be altered by viral and bacterial infections during pregnancy that can cause preterm delivery or result in significant neonatal complications [14,15]. All pregnancy complications mentioned are characterized by systemic inflammation, a known process involved in endothelial dysfunction [16,17,18,19,20,21].

An early diagnosis is crucial for a better outcome of most severe diseases in humans [22,23,24]. Since an early diagnosis of pregnancy complications can significantly improve pregnancy outcome, research is always looking for new specific biomarkers able to allow an early diagnosis of these pregnancy complications [10,25,26,27,28].

It is interesting to note that several natural and synthetic compounds showed beneficial effects in treating pregnancy complications [29,30], suggesting a possible use of these compounds for the treatment/prevention of these diseases. 

Thus, a better knowledge of the mechanisms involved in the regulation of human placenta development under both normal and pathological conditions may bring new perspectives in the treatment of these pregnancy complications.

The aim of this Special Issue is to provide an overview of the physiology and

Pathophysiology of the placenta in order to better understand its development under normal and pathological conditions.
